# The Association Between Nutritional Status and Clinical Outcomes of COVID‐19 Patients in Intensive Care Unit in Vietnam: A Retrospective Study

**DOI:** 10.1002/hsr2.71852

**Published:** 2026-02-26

**Authors:** Hoai Thu Thi Nguyen, Yen Ngoc Ma, Linh Thuy Nguyen, Ngoc Lan Thi Nguyen, Thuy Thi Le, Lan Thi Phuong Dam, Tuan Duc Nguyen

**Affiliations:** ^1^ Department of Biochemistry Hanoi Medical University Hanoi Vietnam; ^2^ Faculty of Medical Laboratory Science Danang University of Medical Technology and Pharmacy Da Nang Vietnam; ^3^ Department of Nutrition and Dietetics, Hanoi Medical University Hospital Hanoi Medical University Hanoi Vietnam; ^4^ Department of Nutrition and Food Safety, Institute for Preventive Medicine and Public Health Hanoi Medical University Hanoi Vietnam; ^5^ Clinical Laboratory Hanoi Medical University Hospital Hanoi Vietnam

**Keywords:** COVID‐19, GLIM, malnutrition, mechanical ventilation, mortality

## Abstract

**Aims:**

This study aimed to assess the nutritional status of COVID‐19 patients within the first 24 h of Intensive Care Unit (ICU) admission and to determine its association with the risk of mechanical ventilation and mortality.

**Methods:**

This retrospective study analyzed electronic medical records from 342 COVID‐19 patients admitted to Hanoi Medical University Hospital, Vietnam, between September 2021 and April 2022. The Global Leadership Initiative on Malnutrition (GLIM) criteria were applied to assess the risk of malnutrition.

**Results:**

According to the GLIM criteria, 77.8% of patients had moderate or severe malnutrition upon ICU admission. Compared with patients without malnutrition, those who were malnourished were significantly older (*p* < 0.001) and had lower BMI, hemoglobin, and red blood cell counts (*p* < 0.05). Univariate logistic regression analysis identified several factors associated with respiratory therapy and mortality among ICU COVID‐19 patients, including age, vaccination status, nutritional status, and BMI. In multivariate logistic regression analysis, malnutrition remained a significant predictor of clinical outcomes. The risk of requiring mechanical ventilation and death were 3.55‐fold and 2.03‐fold higher, respectively, in malnourished patients.

**Conclusion:**

The study revealed a high prevalence of malnutrition among ICU patients with COVID‐19. Furthermore, malnutrition was independently associated with an increased risk of mechanical ventilation and mortality. These findings underscored the importance of early nutritional screening and intervention as integral components of routine management for critically ill COVID‐19 patients.

## 1 Introduction

COVID‐19 is an acute respiratory infection caused by the SARS‐CoV‐2 virus and its variants. The pandemic has posed a major global health leading the World Health Organization (WHO) to declare COVID‐19 a global pandemic on March 11, 2020 [1]. By the end of 2023, COVID‐19 had spread to nearly all countries territories worldwide, resulting in more than 772 million confirmed cases and almost 7 million deaths [2].

Common symptoms of COVID‐19 include fever, shortness of breath, cough, sore throat, and muscle fatigue. While most patients with mild disease did not require hospitalization, those with severe illness often required Intensive Care Unit (ICU) admission and mechanical ventilation and were at increased risk of death [3]. Several factors have been shown to influence disease severity, including age, sex, vaccination status, comorbidities, and nutritional status [4, 5, 6]. Overweight and obesity are known to negatively affected prognosis, as these conditions increase the risk of cardiovascular disease and diabetes. In addition, patients with obesity may experience respiratory complications due to the greater effort required for breathing, impaired respiratory muscle function, and reduced respiratory compliance [7]. On the other hand, several previous studies have reported a high prevalence of malnutrition among COVID‐19 patients and demonstrated that poor nutritional status is associated with prolonged hospitalization, increased rates of ICU admission, and higher mortality [6, 8, 9]. As a result, nutritional management has emerged as a crucial component in the care of patients with COVID‐19 [9].

The Global Leadership Initiative on Malnutrition (GLIM) criteria provide standardized measures for assessing the risk and the presence malnutrition [10]. The GLIM framework has been endorsed by four major international clinical nutrition societies: the European Society for Clinical Nutrition and Metabolism (ESPEN), The American Society for Parenteral and Enteral Nutrition (ASPEN), the Latin American Federation for Parenteral and Enteral Nutrition (FELANPE), and the Parenteral and Enteral Nutrition Society of Asia (PENSA) [11]. A comprehensive meta‐analysis confirmed that the GLIM criteria exhibit high diagnostic accuracy in identifying malnourished patients [12]. Furthermore, GLIM has proven valuable in evaluating the nutritional status of patients with severe COVID‐19 in Vietnam [13]. The GLIM framework includes five diagnostic criteria: two etiological (reduced food intake or assimilation, and inflammation or disease burden) and three phenotypic (unintentional weight loss, low body mass index, and reduced muscle mass). A diagnosis of malnutrion requires at least one phenotypic and one etiological criterion. Due to its simplicity, convenience, and ease of application, the GLIM approach is particularly well‐suited for use in developing countries [10].

By the end of 2023, Vietnam had experienced its fourth wave of COVID‐19 infections, with more than 11 million cases and over 43,000 deaths. However, data on the nutritional status of COVID‐19 patients in Vietnam remain limited [13]. Therefore, this study aimed to assess the nutritional status of COVID‐19 patients within the first 24 h of ICU admission and to determine whether nutritional status was associated with the risk of requiring mechanical ventilation and mortality.

## 2 Methods

A retrospective study was conducted using electronic medical records (EMRs) of 342 COVID‐19 patients admitted to the ICU of X Hospital between September 2021 and April 2022.

Inclusion criteria were: age ≥ 18 years old; confirmed diagnosis of COVID‐19 by real‐time polymerase chain reaction (RT‐PCR); classified as having severe or critical disease according to the COVID‐19 severity classification defined in Decision No. 4689/QD‐BYT dated October 6, 2021, by the Vietnamese Ministry of Health. Patients were categorized as severe if they presented with at least one of the following: respiration rate ≥ 25 breaths per minute; severe shortness of breath; SpO2 < 94% on room air; chest X‐ray or chest computed tomography (CT) scan showing pulmonary infiltrates > 50% or a PaO2/FiO2 ratio between 200 and 300. Patients were categorized as critical if they exhibited at least one of the following: decreased consciousness or coma; respiratory rate ≥ 30 or ≤ 10 breaths per minute; respiratory acidosis; increased respiratory effort; abnormal breathing pattern; requirement for high‐flow nasal cannula therapy or mechanical ventilation; pulse might be fast or slow, with low blood pressure, oliguria, or anuria; chest imaging showing lung infiltrates > 50%; PaO2/FiO2 ratio < 200, with hyperlactatemia (lactate > 2 mmol/L). Exclusion criteria included pregnancy, asymptomatic COVID‐19, mild or moderate disease, and a history of cancer [14].

The sample size was calculated using the estimated proportion formula:

$$n=Z_{{\left(1-\frac{\alpha }{2}\right)}^{2}}x\frac{\left(1-p\right)}{p\varepsilon^{2}}.$$

The study of Mohammadi et al. showed that 67.3% of patients admitted to the ICU had malnutrition. The Z of ($1-\frac{\alpha }{2}$) for a 95% confidence interval was 1.96, ε = 0.1 [15]. Thus, the calculated sample size was 154. Ultimately, data were collected from 342 patients hospitalized during the study period.

Nutritional status was assessedwithin the first 24 h of ICU admission. According to the GLIM criteria, malnutrition was identified based on phenotypic and etiologic components.

The phenotypic criteria included (i) low body mass index (BMI < 18.5 kg/m² for individuals aged < 70 years or < 20 kg/m² for those aged ≥ 70 years); (ii) reduced muscle mass or subcutaneous fat; (iii) non‐volitional weight loss > 5% within the previous 6 months (moderate) or > 10% within the previous 6 months (severe). Reduced muscle mass and subcutaneous fat were determined through anthropometric measurements and clinical examination. The anthropometric indices included calf circumference and mid‐upper arm circumference. Trained healthcare workers assessed muscle mass at specific anatomical sites (temple, clavicle, shoulder, scapula, thenar, thigh/knee, and calf) and evaluated subcutaneous fat loss at the orbital fat pads, buccal fat pads, and triceps.

The etiologic criteria comprised reduced food intake or assimilation and the presence of inflammation or disease burden, as assessed from medical history. Reduced food intake or assimilation was determined by interviewing patients, caregivers, or previous medical staff. When insufficient information was available, dietary intake during the first 24 h of ICU admission was directly observed.

Based on the GLIM criteria, a patient was diagnosed with malnutrition when at least one phenotypic and one etiologic criterion were present [16].

A trained healthcare worker measured each patient's height and weight. For patients able to stand, both measurements were obtained directly within the first 24 h of hospital admission. Standing height was measured using a Seca 213 portable stadiometer (Seca GmbH & Co. KG, Hamburg, Germany) with a precision of 0.1 cm. Weight was measured using a Tanita digital weight scale (Model BC‐758‐WH, Tanita Corporation, Japan). For patients unable to stand upright, weight was measured using an in‐bed scale (Seca 984, Seca GmbH & Co. KG, Hamburg, Germany), and height was estimated using knee height equations specific to the Vietnamese population, in which male's body height = 2.12 * knee height + 59.06 (cm) and female's body height = 2.09 * knee height + 57.37 (cm) [17]. Knee height was measured using a knee height caliper (Cescorf flexible segmometer, model no. 8542031065; Brazil). Body mass index (BMI) was calculated by dividing weight (kg) by height square (m^2^). Weight and height were collected once upon patient admission. BMI classification followed the Asia‐Pacific guidelines: BMI less than 18.5 kg/m^2^ was classified as underweight; between 18.5 and 22.9 kg/m^2^ was classified as normal; ≥ 23 was classified as overweight or obese.

Data were collected from EMRs at two time points: ICU admission and hospital discharge. At admission, data gathered general information such as age, sex, comorbidities and medical history, vaccination status, height, weight, and BMI, nutritional assessment (based on GLIM), and biomedical parameters including albumins, ferritin, creatinine, AST, ALT, and a complete blood count. At discharge, data included type of respiratory therapy (mechanical ventilation vs. non‐ventilation) and clinical outcomes (survival vs. non‐survival).

### 2.1 Statistical Analysis

Data were entered using EpiData version 3.1 and analyzed with Stata/BE 17.0 (StataCorp LLC, College Station, TX, USA). Data distributions were assessed using the skewness and kurtosis normality tests in Stata. Categorical variables were summarized as frequencies and percentages, and continuous variables were expressed as means ± standard deviations (SD). For between‐group comparisons, the t‐test was applied for normally distributed continuous variables, and the Mann–Whitney U test was used for non‐normally distributed data. The Chi‐square (χ^2^) test was used to compare categorical variables, and the Chi‐square (χ^2^) test for trend was applied to ordinal variables when assumptions were met.

Both univariate and multivariate logistic regression analyzes were performed to identify background variables (independent variables) significantly associated with respiratory therapy and clinical outcomes (dependent variables). The dependent variable was binary, while independent variables included categorical, ordinal, and interval/ratio. Variables with a *p*‐value < 0.05 in the univariate analysis were included in the multivariate model. To account for potential confounders, a multivariate logistic regression model was constructed to estimate adjusted odds ratios (ORs) with 95% confidence intervals (CIs). Statistical significance was considered at *p*‐value < 0.05.

### 2.2 Ethical Approval Statement

This study adhered to the Strengthening the Reporting of Observational Studies in Epidemiology (STROBE) guidelines (Supporting Information File S1). Ethical approval was obtained from the Institutional Review Board (IRB) of Ethics in Biomedical Research designated by the code 546/GCN‐HÐÐÐNCYSH‐ÐHYHN on 07 July, 2021, and code 846/GCN‐ HÐÐÐNCYSH‐ÐHYHN (7 June, 2023). Written informed consent was obtained from all patients or legal guardians at the time of hospital admission. The study procedures did not interfere with patient diagnosis or treatment. Data collection was performed using EMRs with prior authorization from the hospital. All research activities were conducted exclusively on anonymized and encrypted patient data provided by the medical facility. Patient confidentiality was strictly maintained, and all data usage complied with relevant data protection and privacy regulations.

## 3 Results

A total of 342 COVID‐19 patients admitted to the ICU were included in the analysis. Among them, 206 patients required mechanical ventilation, and 152 patients died. The mean age of the participants was 71.1 ± 16.1 years (range: 18‐103 years), and 73.1% were aged over 65 years. Mortality was significantly associated with age: 81.6% of deaths occurred in patients aged ≥ 65 years, compared with 18.4% in those < 65 years (*p* = *0.002*). The mean age was also significantly higher among non‐survivors(74.5 vs. 68.4 years; *p* < 0.001). The sex distribution was 52.1% male and 47.9% female. A substantial proportion of patients presented with underlying comorbidities, including heart failure (13.2%), chronic kidney disease (12.9%), and hypertension (55.9%). The proportion of unvaccinated participants was 55.3%, which was significantly higher among both the mechanical ventilation group (59.7% vs. 40.3%; *p* = 0.042) and the mortality group (63.8% vs. 36.2%; *p* = 0.004) (Table 1).

**Table 1:** Characteristics of the study participants.

Variables		Respiratory therapy			Clinical outcomes		
	Total (*n* = 342)	Non‐mechanical ventilation (*n* = 136)	Mechanical ventilation (*n* = 206)	*p* value	Survivors (*n* = 190)	Non‐Survivors (*n* = 152)	*p* value
Age				0.81**			< 0.001**
Mean ± SD	71.1 ± 16.1	70.8 ± 16.4	71.3 ± 15.9		68.4 ± 16.8	74.5 ± 14.4	
(min–max)	(18–103)	(20–100)	(18–103)		(20–100)	(18–103)	
Age group, *n* (*%*)				0.40*			0.002*
< 65 years	92 (26.9)	40 (11.7)	52 (15.2)		64 (18.7)	28 (8.2)	
≥ 65 years	250 (73.1)	96 (28.1)	154 (45.0)		126 (36.8)	124 (36.3)	
Sex, *n* (*%*)				0.69*			0.37*
Male	178 (52.1)	69 (20.2)	109 (31.9)		103 (30.1)	75 (22.1)	
Female	164 (47.9)	67 (19.6)	97 (28.3)		87 (25.4)	77 (22.5)	
Other comorbidities, *n* (*%*)	280 (81.8)	116 (33.9)	164 (47.9)		149 (43.6)	131 (38.2)	
Hypertension	191 (55.9)	82 (24.0)	109 (31.9)	0.18*	104 (30.4)	87 (25.5)	0.64*
Heart failure	45 (13.2)	13 (3.8)	32 (9.4)	0.11*	20 (5.8)	25 (7.4)	0.11*
CKD	44 (12.9)	21 (6.1)	23 (6.8)	0.25*	25 (7.3)	19 (5.6)	0.86*
COVID‐19 Vaccine, *n* (*%*)				0.04*			0.004*
Vaccinated	153 (44.7)	70 (20.5)	83 (24.2)		98 (28.7)	55 (16.0)	
Non‐vaccinated	189 (55.3)	66 (19.3)	123 (36.0)		92 (26.9)	97 (28.4)	

Abbreviations: SD, standard deviation.

* χ^2^ test.

** t‐test; CKD: Chronic Kidney Disease.

According to the GLIM criteria, 77.8% of the participants were classified as having moderate to severe malnutrition with 47.1% having moderate and 30.7% having severe malnutrition. The mean hemoglobin and red blood cell (RBC) counts were significantly higher in the non‐malnourished group than in the malnourished group (*p* = 0.023 and *p* = 0.040, respectively). The average age of the malnourished group was significantly greater than that of the non‐malnourished group (73.4 ± 15.4 vs. 63.1 ± 15.9 years; *p* < 0.001). The difference in the proportion of participants over 65 years between the two groups was also statistically significant (*p* < 0.001). No significant differences were observed between the two groups regarding serum albumin, creatinine, liver function indicators, or comorbidities (Table 2).

**Table 2:** The association between study participants' characteristics and nutritional status according to GLIM.

Variables	GLIM assessment (*n*= 342)		*p* value
	Non‐malnutrition (*n* = 76)	Malnutrition (*n* = 266)	
Age group, *n* (%)	38 (11.1)	54 (15.8)	< 0.001*
< 65	38 (11.1)	212 (62.0)	
≥ 65			
Sex *n* (%)	45 (13.2)	133 (38.9)	0.16*
Male	31 (9.0)	133 (38.9)	
Female			
BMI (*n* = 342), *n* (%)	0 (0)	46 (13.5)	< 0.001*
< 18.5	21 (6.1)	141 (41.2)	
18.5–22.9	55 (16.1)	79 (23.1)	
≥ 23			
Other comorbidities, *n* (%)	55 (16.1)	225 (65.8)	
Hypertension	40 (11.7)	151 (44.1)	0.55*
Heart failure	7 (2.0)	38 (11.1)	0.24*
Chronic kidney disease	8 (2.4)	36 (10.6)	0.46*
Age (*n* = 342) (Mean ± SD)	63.1 ± 15.9	73.4 ± 15.4	< 0.001**
BMI (*n* = 342) (Mean ± SD)	24.5 ± 2.8	21.7 ± 3.4	< 0.001**
RBC [T/L] (*n *= 342) (Mean ± SD)	4.55 ± 0.9	4.32 ± 0.8	0.04**
Hemoglobin [g/L] (*n *= 342) (Mean ± SD)	133.9 ± 24.5	126.7 ± 23.8	0.02**
Ferritin [ng/mL] (*n *= 263) (Mean ± SD)	1347.6 ± 557.4	1300.4 ± 602.5	0.59**
Albumin [g/L] (*n *= 242) (Mean ± SD)	30.4 ± 4.8	28.9 ± 5.0	0.07**
Creatinine [µmol/L] (*n* = 328)	74	81	0.30**
Median (IQR)	(59–101)	(59–117)	
AST [UI/L] (*n* = 308)	52	50.5	0.79**
Median (IQR)	(35–75)	(32–79.5)	
ALT [UI/L] (*n* = 307)	33.5	33	0.47**
Median (IQR)	(22.5–62)	(22–53)	

Abbreviations: ALT, alanine aminotransferase; AST, aspartate aminotransferase; BMI, body mass index; GLIM, global leadership initiative on malnutrition; RBC, red blood cell count.

* χ^2^ test.

** t‐test; SD: standard deviation; IQR: Interquartile range.

Nutritional status was significantly associated with both respiratory therapy and clinical outcomes among ICU COVID‐19 patients. The mortality rate was markedly higher in the malnourished group compared with the non‐malnourished group (49.6% vs. 26.3%; *p* < 0.01), and the use of mechanical ventilation was significantly more frequent (67.7% vs. 34.2%; *p* < 0.01). Approximately 80% of patients in both the mechanical ventilation and mortality cohorts reported a 50%–75% reduction in their usual daily nutritional intake prior to ICU admission. Significant differences were also found between the ventilated and non‐ventilated groups in terms of non‐volitional weight loss, loss of muscle mass or subcutaneous fat, and serum albumin levels (*p* < 0.05) (Table 3).

**Table 3:** Characteristics of nutritional status among study participants groups.

Nutritional parameter evaluation	Total (*n* = 342)	Respiratory therapy		*p* value*	Clinical outcomes		*p* value*
		Non‐ mechanical ventilation (*n* = 136)	Mechanical ventilation (*n* = 206)		Survivors (*n* = 190)	Non‐Survivors (*n* = 152)	
GLIM *n* (%)							
No malnutrition	76 (22.2)	50 (14.6)	26 (7.6)	< 0.01	56 (16.4)	20 (5.8)	< 0.01
Moderate malnutrition	161 (47.1)	51 (14.9)	110 (32.1)		85 (24.9)	76 (22.2)	
Severe malnutrition	105 (30.7)	35 (10.2)	70 (20.6)		49 (14.3)	56 (16.4)	
BMI group *n* (%)
BMI < 18.5	46 (13.4)	21 (6.1)	25 (7.3)	0.21	26 (7.6)	20 (5.8)	0.09
18.5 ≤ BMI ≤ 22.9	162 (47.4)	55 (16.1)	107 (31.3)		79 (23.1)	83 (24.3)	
BMI > 23	134 (39.2)	60 (17.5)	74 (21.7)		85 (24.9)	49 (14.3)	
Non‐volitional weight loss *n* (%)							
None	217 (63.5)	103 (30.1)	114 (33.3)	< 0.05	120 (35.1)	97 (28.4)	0.92
Moderate to severe	125 (36.5)	33 (9.6)	92 (27.0)		70 (20.5)	55 (16.0)	
Loss of muscle mass/subcutaneous fat, *n* (%)							
Mild to moderate	181 (52.9)	83 (24.3)	98 (28.7)	0.03	105 (30.7)	76 (22.2)	0.53
Severe	137 (40.1)	43 (12.6)	94 (27.5)		71 (20.8)	66 (19.3)	
Normal	24 (7.0)	10 (2.9)	14 (4.0)		14 (4.1)	10 (2.9)	
Reduced food intake *n* (%)							
Normal				< 0.01			< 0.01
< 50% of normal intake	103 (30.1)	64 (18.7)	39 (11.4)		69 (20.2)	34 (9.9)	
< 75% of normal intake	199 (58.2)	68 (19.9)	131 (38.3)		105 (30.7)	94 (27.5)	
	40 (11.7)	4 (1.2)	36 (10.5)		16 (4.7)	24 (7.0)	

Abbreviations: BMI: body mass index; GLIM, global leadership initiative on malnutrition.

* χ^2^ test.

In the univariate logistic regression models, several factors were significantly associated with both respiratory support requirements and mortality among ICU COVID‐19 patients, including age, vaccination status, nutritional status, and BMI (Table 4). Malnutrition was independently associated with increased likelihood of requiring mechanical ventilation (OR = 3.55; 95% CI: 1.93–6.55; *p* < 0.001) as well as higher mortality (OR = 2.03; 95% CI: 1.09–3.78; *p* = 0.026) in ICU COVID‐19 patients. Additionally, vaccination status was also identified as an independent predictor of mortality (OR = 1.66; 95% CI: 1.04–2.67; *p* = 0.035) (Table 5).

**Table 4:** Characteristics of sub‐clinical indicators among groups of study participants.

Nutritional sub‐clinical biomarker assessment	*n*	Total	Respiratory therapy		*p* value*	Clinical outcomes		*p* value*
			Non‐ mechanical ventilation (*n* = 136)	Mechanical ventilation (*n* = 206)		Survivors (*n* = 190)	Non‐survivors (*n* = 152)	
RBC [T/L] Mean ± SD (Min–Max)	342	4.4 ± 0.8 (1–6.8)	4.5 ± 0.8 (1–6.7)	4.3 ± 0.9 (1.4–6.8)	0.16	4.4 ± 0.9 (1–6.7)	4.3 ± 0.8 (1.5–6.8)	0.18
Hemoglobin [g/L] Mean ± SD (Min–Max)	342	128.4 ± 24.1 (30–179)	130.7 ± 23.5 (30–179)	126.9 ± 24.4 (45–175)	0.16	129.5 ± 24.3 (30–179)	126.9 ± 23.9 (50–171)	0.32
Ferritin [ng/mL] Mean ± SD (Min–Max)	263	1311.3 ± 541.6 (31.3–2001)	1313.7 ± 586.2 (31.3–2001)	1309.6 ± 597.4 (35.8–2001)	0.96	1288.1 ± 579.9 (31.3–2001)	1341.2 ± 607.5 (35.8–2001)	0.47
Albumin [g/L] Mean ± SD (Min–Max)	242	29.1 ± 4.97 (13.9–42.4)	30.9 ± 5.0 (14.2–42.4)	28.4 ± 4.8 (13.9–40.2)	< 0.001	29.6 ± 4.9 (14.2–42.4)	28.7 ± 5.0 (13.9–40.2)	0.18
Creatinine [µmol/L] (*n* = 328) Median (IQR)		79.5 (59–114)	76 (18–629)	83 (60–124)	0.14	76 (59–107)	82 (59–122)	0.36
AST [UI/L] (*n* = 308) Median (IQR)		51 (32.5–78.5)	46 (31–71)	54.75 (35–88)	0.47	76 (59–107)	82 (59–122)	*0.03*
ALT [UI/L] (*n* = 307) Median (IQR)		33 (22–55)	33 (22–55)	34.5 (21.5–55)	0.57	32 (21–53)	35 (22–57)	0.42

Abbreviations: ALT, alanine aminotransferase; AST, aspartate aminotransferase; RBC, red blood cell count.

* t‐test.

**Table 5:** Univariate and multivariate logistic regression models for predictors of mechanical ventilation.

Variables	Univariate			Multivariate		
	OR	95% CI	*p*‐value	OR	95% CI	*p* value
Age groups (*n* = 342) (≥ 65 vs. < 65)	1.23	0.76–2.00	0.40	0.96	0.53–1.73	0.89
COVID‐19 vaccine status (*n* = 342) (Non‐vaccinated vs. Vaccinated)	1.57	1.01–2.43	0.04	1.49	0.92–2.43	0.11
Sex (*n* = 342) (Male vs. Female)	1.09	0.71–1.68	0.69	0.82	0.51–1.33	0.42
Nutritional status (GLIM) (*n* = 342) (Malnutrition vs. Non‐malnutrition)	4.03	2.35–6.90	< 0.001	3.55	1.93–6.55	*< 0.001*
BMI (*n* = 342) (BMI ≥ 23 kg/m^2^ vs. < 23 kg/m^2^)	0.71	0.46–1.11	0.13	1.10	0.64–1.89	0.72
Hypertension (*n* = 191) (Yes vs. No)	0.71	0.45–1.12	0.14	0.83	0.43–1.61	0.59
RBC [T/L] (*n* = 342)	1.46	0.92–2.30	0.10			
Hemoglobin [g/L] (*n* = 342)	1.38	0.87–2.20	0.17			
Ferritin [ng/mL] (*n* = 263)	0.49	0.13–1.86	0.30			
Albumin [g/L] (*n* = 242)	1.26	0.58–2.70	0.56			
Creatinine [µmol/L] (*n* = 328)	1.54	0.98–2.42	0.06			
AST [UI/L] (*n* = 308)	1.52	0.94–2.47	0.09			
ALT [UI/L] (*n* = 307)	1.12	0.73–1.73	0.60			

Abbreviations: ALT, alanine aminotransferase; AST, aspartate aminotransferase; BMI, body mass index; GLIM, global leadership initiative on malnutrition; RBC, red blood cell count.

## 4 Discussion

COVID‐19 patients are particularly vulnerable to malnutrition due to the metabolic stress associated with fever, tissue repair, infection control, and the management of disease‐related complications. Additionally, these patients often experience symptoms such as loss of taste and appetite, and digestive disturbances, particularly those with severe illness, under quarantine, or receiving intensive care in overloaded ICUs. Such conditions made maintaining adequate nutritional intake extremely challenging [18]. Most COVID‐19 treatment guidelines recommend assessing nutritional status upon hospital admission using validated screening tools, including the Malnutrition Universal Screening Tool (MUST), Nutrition Risk Screening‐2002 (NRS‐2002), Mini Nutritional Assessment (MNA), MNA‐Short Form (MNA‐sf), Nutritional Risk Index (NRI), and the GLIM [13, 19]. However, each tool has its own advantages and disadvantages, and there is currently no universally accepted “gold standard” for assessing nutritional risk in these patients [20]. In this study, we used the GLIM criteria because they are specific, simple, and practical for use in hospital settings. The GLIM diagnostic framework requires at least one etiological and one phenotypic criterion, with multiple options under each domain to facilitate data collection, especially useful in severely ill, quarantined patients who often lack caregiver support [10, 21]. In our study, the prevalence of moderate to severe malnutrition was high (77.8%). This can be explained by the fact that our participants were among the most critically ill COVID‐19 patients admitted to a specialized COVID‐19 hospital in Northern Vietnam. Furthermore, the majority of patients had chronic comorbidities such as hypertension and chronic kidney disease, both of which often required dietary restrictions. These comorbidities likely contributed to reduced food intake and subsequent malnutrition‐related immunodeficiency [22].

Nutrition plays a crucial role in maintaining overall health. Insufficient intake of essential nutrients can impair growth, weaken the immune system, exacerbate metabolic stress, and trigger a systemic inflammatory responses. Prolonged malnutrition may lead to organ failure, increased susceptibility to infection, and reduce chances of recovery [13, 23]. Several previous studies reported a high prevalence of malnutrition and its adverse impact on the prognosis of COVID‐19 patients, including longer hospital stays, higher ICU transfer rates, and increased mortality [6, 8, 9]. However, unlike most of these studies that investigated patients across all disease severities [24], our study specifically focused on severe COVID‐19 cases admitted to the ICU. Both univariate and multivariate logistic regression analyzes confirmed that malnutrition remained a significant contributing factor to the clinical outcome of ICU COVID‐19 patients (Table 6). The likelihood of requiring mechanical ventilation was over three times higher (OR = 3.55; 95% CI: 1.93–6.55; *p* < 0.001), and the risk of death was more than twice as high (OR = 2.03; 95% CI: 1.09–3.78; *p* = 0.026) in malnourished patients. These findings are consistent with the study by Wei et al., who used the Controlling Nutritional Status (CONUT) score to predict mortality COVID‐19 patients, reporting a 1.736‐fold higher risk of death in those classified as malnourished [25]. In addition to malnutrition, overweight and obesity were also associated with mortality among ICU COVID‐19 patients in our univariate analysis (Table 5). Similar findings have been reported previously [4], suggesting that excess adiposity contributes to a pro‐inflammatory state through the secretion of adipokines, chemokines, and cytokines such as tumor necrosis factor‐α, interleukin‐6, and interleukin‐1β from visceral adipose tissue. This inflammation alters immune responses and promotes cellular dysfunction. Furthermore, obesity‐related changes in leukocyte populations, including increased macrophages infiltration in adipose tissue, enhanced granulocyte and natural killer cell proliferation, and impaired helper T and B cell function, collectively weaken host defense mechanisms. These factors likely contribute to an ineffective immune response against infections, including SARS‐CoV‐2 [26].

**Table 6:** Univariate and multivariate logistic regression models for predictors of mortality.

Variables	Univariate			Multivariate		
	OR	95% CI	*p* value	OR	95% CI	*p* value
Age groups (*n* = 342) (≥ 65 vs. < 65)	2.25	1.35–3.74	0.002	1.419	0.84–2.66	0.17
COVID‐19 vaccine status (*n* = 342) (Non‐vaccinated vs. Vaccinated)	1.88	1.21–2.91	0.005	1.66	1.04–2.67	0.04
Sex (*n* = 342) (Male vs. Female)	0.82	0.54–1.26	0.37	1.15	0.72–1.84	0.55
Nutritional status (GLIM) (*n* = 342) (Malnutrition *vs*. Non‐malnutrition)	2.76	1.57–4.85	< 0.001	2.03	1.09–3.78	0.03
BMI (*n* = 342) (BMI ≥ 23 kg/m^2^ vs. < 23 kg/m^2^)	0.59	0.38–0.92	0.02	0.76	0.45–1.28	0.31
Hypertension (*n* = 191) (Yes vs. No)	1.05	0.67–1.63	0.84	1.22	0.66–2.28	0.52
RBC [T/L] (*n* = 342)	1.23	0.79–1.91	0.37			
Hemoglobin [g/L] (*n* = 342)	1.48	0.95–2.32	0.08			
Ferritin [ng/mL] (*n* = 263)	0.56	0.17–1.80	0.33			
Albumin [g/L] (*n* = 242)	0.73	0.34–1.56	0.41			
Creatinine [µmol/L] (*n* = 328)	1.08	0.69–1.69	0.74			
AST [UI/L] (*n* = 308)	1.58	0.97–2.60	0.07			
ALT [UI/L] (*n* = 307)	0.85	0.55–1.30	0.46			

Abbreviations: ALT, alanine aminotransferase; AST, aspartate aminotransferase; BMI, body mass index; GLIM, global leadership initiative on malnutrition; RBC, red blood cell count.

Vaccination has been one of the most effective measures against COVID‐19, with numerous studies demonstrating its positive impact in reducing hospitalization rates, mitigating disease severity, and lowering mortality [27, 28]. Consistent with these findings, our study also revealed that vaccination played a significant role in the clinical outcome of ICU COVID‐19 patients (Tables 4 and 5). Even after adjusting for other variables, unvaccinated individuals had a 1.66‐fold higher risk of mortality compared with vaccinated patients (Table 5). Additionally, age has long been recognized as a major determinant of COVID‐19 severity [4, 5, 6]. Our univariate logistic regression analysis similarly indicated that older age was associated with higher mortality among ICU COVID‐19 patients; however, this relationship did not remain significant in the multivariate model (Table 5). Advanced age is typically accompanied by multiple physiological changes, including a higher prevalence of comorbidities, impaired immune responses, reduced organ function, and diminished vaccine efficacy [29, 30]. Therefore, age might be better considered a secondary factor rather than an independent prognostic indicator of poor outcomes. Importantly, our findings revealed a close relationship between advanced age and poor nutritional status, particularly among patients aged 65 years and older (Table 2). This observation aligned with previous studies on similar topics [18, 20, 21]. Such vulnerability is not unique to COVID‐19 but is commonly observed among elderly populations, who are more susceptible to malnutrition due to age‐related physiological decline, limited dietary intake, and multiple chronic comorbidities. These results highlighted the complex, multifactorial nature of COVID‐19 prognosis and underscored the importance of incorporating comprehensive nutritional and clinical assessments into the management of critical ill patients.

This study had some limitations. First, obtaining comprehensive data for all variables was not always feasible because many participants were critically ill. Bioelectrical impedance analysis (BIA) effectively provides a more precise assessment of body composition and helps differentiate between true malnutrition and conditions such as fluid overload. Financial and equipment limitations, along with Vietnam's epidemic prevention policies, made it impractical to implement BIA devices in a temporary field hospital during the peak of the COVID‐19 pandemic. Second, the retrospective design of this study prevented the establishment of a causal relationship between nutritional status, nutritional intervention, and patient outcomes. Finally, as a single‐center study, the findings might have limited generalizability. Future prospective, multi‐center studies are warranted to validate these results and overcome the aforementioned limitations.

This research underscored the high prevalence of malnutrition among COVID‐19 patients admitted to the ICU. Additionally, malnutrition was independently associated with an increased likelihood of requiring mechanical ventilation and a higher mortality. These findings emphasize the importance of early nutritional screening and intervention to mitigate the risk of malnutrition and improve clinical outcomes in critically ill COVID‐19 patients.

## 5 Conclusion

The study revealed a high prevalence of malnutrition among ICU patients with COVID‐19. Furthermore, malnutrition was independently associated with an increased risk of mechanical ventilation and mortality. These findings underscored the importance of early nutritional screening and intervention as integral components of routine management for critically ill COVID‐19 patients.

## Author Contributions

**Tuan Duc Nguyen, Ngoc Lan Thi Nguyen, Linh Thuy Nguyen:** conceptualization, data curation, methodology, investigation, validation, formal analysis, supervision, funding acquisition, visualization, project administration, resources, writing – review. **Hoai Thu Thi Nguyen:** data curation, investigation, methodology, project administration, resources, visualization, writing – original draft, writing – review and editing. **Yen Ngoc Ma:** data curation, formal analysis, writing – original draft, writing – review and editing. **Thuy Thi Le:** data curation, methodology, resources, software, writing – original draft, writing – review. **Lan Thi Phuong Dam:** writing – original draft, writing – review and editing. All authors have read and approved the final version of the article.

## Conflicts of Interest

The authors declare no conflicts of interest.

## Transparency Statement

The lead author Tuan Duc Nguyen affirms that this article is an honest, accurate, and transparent account of the study being reported; that no important aspects of the study have been omitted; and that any discrepancies from the study as planned (and, if relevant, registered) have been explained.

## Supporting information

Supplementary S1_STROBE_checklist_HSR‐2025‐02‐0588.

## Data Availability

The raw data supporting the conclusions of this article are available from the authors upon reasonable request.
